# Intermolecular Non-Bonded Interactions from Machine Learning Datasets

**DOI:** 10.3390/molecules28237900

**Published:** 2023-12-01

**Authors:** Jia-An Chen, Sheng D. Chao

**Affiliations:** 1 Institute of Applied Mechanics, National Taiwan University, Taipei 106, Taiwan; r08543055@ntu.edu.tw; 2 Center for Quantum Science and Engineering, National Taiwan University, Taipei 106, Taiwan

**Keywords:** non-bonded interactions, machine learning potentials, symmetry adapted perturbation theory, quantum chemistry datasets, artificial intelligence

## Abstract

Accurate determination of intermolecular non-covalent-bonded or non-bonded interactions is the key to potentially useful molecular dynamics simulations of polymer systems. However, it is challenging to balance both the accuracy and computational cost in force field modelling. One of the main difficulties is properly representing the calculated energy data as a continuous force function. In this paper, we employ well-developed machine learning techniques to construct a general purpose intermolecular non-bonded interaction force field for organic polymers. The original ab initio dataset SOFG-31 was calculated by us and has been well documented, and here we use it as our training set. The CLIFF kernel type machine learning scheme is used for predicting the interaction energies of heterodimers selected from the SOFG-31 dataset. Our test results show that the overall errors are well below the chemical accuracy of about 1 kcal/mol, thus demonstrating the promising feasibility of machine learning techniques in force field modelling.

## 1. Introduction

Accurate determination of intermolecular non-covalent-bonded or non-bonded interactions is the key to potentially useful molecular dynamics simulations of polymer systems, with the hope to model biological macromolecules. However, it is really a daunting task to calculate these relatively weak interaction energies accurately, not to mention modelling them into smooth potential functions, called force fields, for use in molecular dynamics simulations. Starting from empirical data, together with statistical methods, most traditional force field constructions rely on choosing a definite function formula to model the observed discrete data available. This approach is advantageous in obtaining in short time a first understanding of the system studied, but usually the predictive power is weak due to errors in the empirical data and intrinsic constraints on the chosen function forms. Not only does the original dataset bear empirical errors, and is usually restricted in its applicability to the training set, but the function formula chosen may not properly represent the force field. To solve the first problem, we have in the past decade calculated the interaction energies of small organic molecular dimers using quantum chemistry methods and collected the data into well-organized datasets. These ab initio datasets are in principle the working standard with the desired chemical accuracy. The second issue, namely how to properly represent the calculated energy data as a continuous force function, is the point where data-based techniques come into play in this subfield of computational molecular science.

Recent machine learning (ML) and deep learning (DL) techniques have given great impetus to advancing traditional non-linear regression algorithms to a remarkable new level, with applications in almost all scientific research fields [1,2,3,4,5,6,7,8,9,10]. Many ML and DL algorithms have been well developed and tested, significantly including artificial neural networks (ANNs), graph convolutional networks (GCNs), and kernel-ridge regression (KRR), among others [11,12,13,14]. These algorithms are particularly suitable to use in current computer hardware systems, such as graphics processing units. Without a doubt, these artificial intelligence (AI) generated methods are very powerful tools, used in a wide range of diverse fields. The main scheme of a data-driven analysis relies heavily on the ability of the algorithm to faithfully estimate the features in unknown domains by quick and quantitative regression of a large amount of training data. Although there is a rigorous mathematical background behind these artificial intelligence (AI) methods [15,16,17,18,19,20,21], in practice, most studies still rely on the usual trial-and-error cycle of training and testing. Therefore, the training data input into a ML algorithm plays the most important role in its potential success, including the ultimate goal of finding a non-analytical representation of the main features of the dataset studied.

To avoid useless outcomes from immaterial inputs, one must keep alert in monitoring the data propagation processes involved in a “black box” algorithm and judge the results by employing human knowledge [22]. Indeed, these upsurging ML algorithms give hope to solving many intractable non-linear regression problems, and if successful, they may offer promising potential for use in any data-based research field [23,24,25,26,27,28,29,30,31]. One of the most applied research fields in macromolecular chemistry, materials discovery and drug candidate screening, must accurately characterize the non-covalent-bonded or non-bonded interactions involved among highly diverse and heterogeneous material, medical, and physiological environments [32,33,34]. These faint but numerous “secondary” interactions play a crucial role in determining the overall morphology of macromolecules such as proteins and DNAs. The unknown analytical, if any, functions of the non-bonded interactions are the target object that one intends to find in theoretical chemistry studies. For this specific purpose, recently, machine learning potentials have become more and more popular, although their fundamental mechanism for obtaining well reported results have not been systematically tested or universally accepted.

The past two decades have witnessed a remarkable advancement of using ab initio data to build ML potentials in conventional force field (FF) constructions [35,36,37,38,39,40,41,42,43,44,45]. In particular, for small molecular dimers (roughly less than 50 atoms involved), highly correlated first-principles quantum chemistry methods, such as coupled-cluster (CC) theory, have been routinely calculated, with data collected that can be used to provide benchmark accuracy, including ab initio data to calibrate other less accurate but more efficient calculation methods, such as the density functional theory (DFT). However, for larger dimers or macromolecules, a balance between accuracy and speed must be achieved to obtain meaningful predictions of chemical structures and the kinetics of macromolecules. For this purpose, alternative symmetry adapted perturbation theory- (SAPT) calculated intermolecular interaction energies have become more and more popular [46,47,48,49,50,51,52,53,54,55,56]. SAPT can be used to obtain direct interaction energies without complications from the basis set superposition error (BSSE) problems involved in basis set expansion methods. In addition, the theory, by its construction, separates the overall interaction energy into four physically meaningful terms: electrostatic, exchange–repulsion, induction/polarization, and dispersion components. These SAPT-calculated energy data serve as a very attractive candidate for use in drug binding or macromolecular recognition, mainly because of their acceptable accuracy levels and the reasonable scaling in computational cost, in particular in the moderate approximation form called the SAPT0. This method has been popularly used in recent studies and has achieved a very promising level of success in modeling molecular segments or motifs of macromolecules. For example, a recently published DES370K dataset [57], as its name indicates, has collected some tens of thousands of dimeric interaction energies in a reasonable calculation timespan. Therefore, one can now routinely generate moderate accuracy data on a large scale using the SAPT0. Once the data is available, the next task is to model them, with the goal of obtaining universal force fields.

Traditional force field modelling involves complicated non-linear regression schemes on a wide range of potential energy data. Very often it is difficult to determine uniquely a set of proper parameters with a given functional form of representation. As the data increase in amount, the corresponding parameters also increase in number, sometimes even more than the data; this causes an overfitting problem. Recent machine learning techniques can be used to solve this problem, so in this paper we perform our first attempt at modelling the previously home-constructed SOFG-31 dataset [58,59] in order to test the feasibility of the recently proposed CLIFF kernel type ML algorithm [60].

This paper is organized into four sections. In Section 2, we show our main results and discussion. The methodology details are shown in Section 3. The last section concludes this work. 

## 2. Results and Discussion 

### 2.1. Employing the SOFG-31 Dataset as the Fitting Dataset

Any data-based studies count on properly prepared datasets. Generally there are two schools of thought about how to prepare input interaction energy datasets. One way is to obtain the energies by using less accurate but more efficient methods. For example, using empirical force fields to perform molecular dynamics simulations yields randomly distributed molecular structures and the corresponding energies for these structures. The datasets obtained in this way are often used together with supervised ML algorithms. The other is to systematically calculate the energies by using high accuracy quantum chemistry methods for well-organized molecular types and structures. The datasets obtained in this way can bear many important molecular features in a diverse domain of sample data space, hence they are usually used together with semi-supervised ML algorithms. 

In this paper, we prepare our SOFG-31 datasets using the second method, similar to the original CLIFF0 datasets and the Des370k dataset. The SOFG-31 dataset was recently released by our lab and contains ab initio interaction energy data calculated via a minimum level quantum chemistry standard method, namely the SAPT method (see Section 3). The SOFG-31 dataset contains a total of 31 small organic functional groups across eight common classes: alkanes, alkenes, alkynes, alcohols, aldehydes, ketones, carboxylic acids, and amides. The dimers within this dataset are exclusively composed of homodimers in equilibrium state, as shown in Table 1. Notice that these data are well-organized according to the specific organic functional groups, to which typical chemical features of the molecular series are assigned. For example, the binding energy contribution of the hydrogen bond interaction component increases across the groups from the alkanes to the amides. Therefore, one expects to utilize these features in the training and learning processes. Indeed, we built the training data by strictly following the known energy patterns of these group classifications. Recently, we also extended the SOFG-31 dataset to the SOFG-31-heterodimer dataset by including 269 heterodimers selected from the SOFG-31 dataset. For the first case, we use the SOFG-31 dataset as the training set and the SOFG-31-heterodimer dataset as the test set.

**Figure 1:**
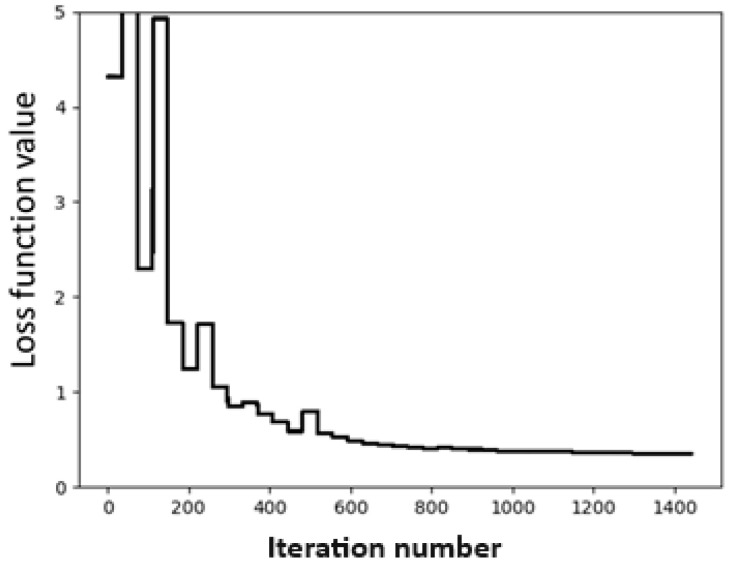
Convergence of the loss function during the fitting process.

We employ the standard Broyden–Fletcher–Goldfarb–Shanno (BFGS) method in the optimization process, where a multi-target loss function *L* is used to minimize the mean square errors (MSE) of both the SAPT component energies and the total energy, with a distributive parameter *γ* set to 0.4, as shown in Equation (1)

(1) $$L=(1-\gamma )\mathrm{MSE}(E_{total})+\gamma \sum_{i\in C}\mathrm{MSE}(E_{i})$$

where *C* represents the set of the four SAPT components. The convergence test is shown in Figure 1, where the abscissa denotes the number of iterations, and the ordinate represents the values of the loss function in kcal/mol. From Figure 1, we can see that the fitting process converges very quickly after the iteration number exceeds 300.

The set of the optimized CLIFF parameters is shown in Table 2. We note that there are many blanks in this table compared to the published table using the CLIFF0 dataset. This is because the dimer data in the SOFG-31 dataset do not contain the designated atomic species. However, we test this set of parameters with the SOFG-31-heterodimer dataset to confirm that using the SOFG-31 training set correctly predicts the intermolecular interaction energies of the SOFG-31-heterodimer dataset.

#### Predicted Results and Comparison to the SOFG-31-Heterodimer Dataset

As a first test of the ML potential data, we employ the parameters derived from the SOFG-31 dataset, the training dataset, to predict the energy data within the SOFG-31- heterodimer dataset (Table 3). Here, we compile the data using three length measures: the mean absolute error (MAE), the root mean square error (RMSE), and the maximum deviation (MAX) for each SAPT energy component. As can be seen in Table 3, both the length order 1 and order 2 (L1 and L2) error measures are within about 1 kcal/mol, which indicates the feasibility of the trained ML potential. The MAX values are well controlled, albeit a large value appears to be associated with the exchange repulsion energy, where the sampling points are rare. 

**Figure 2:**
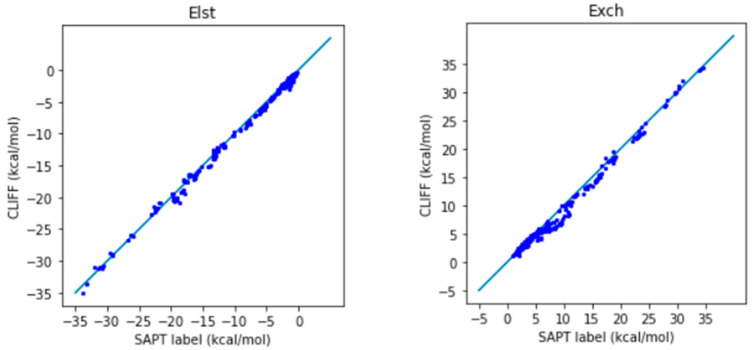

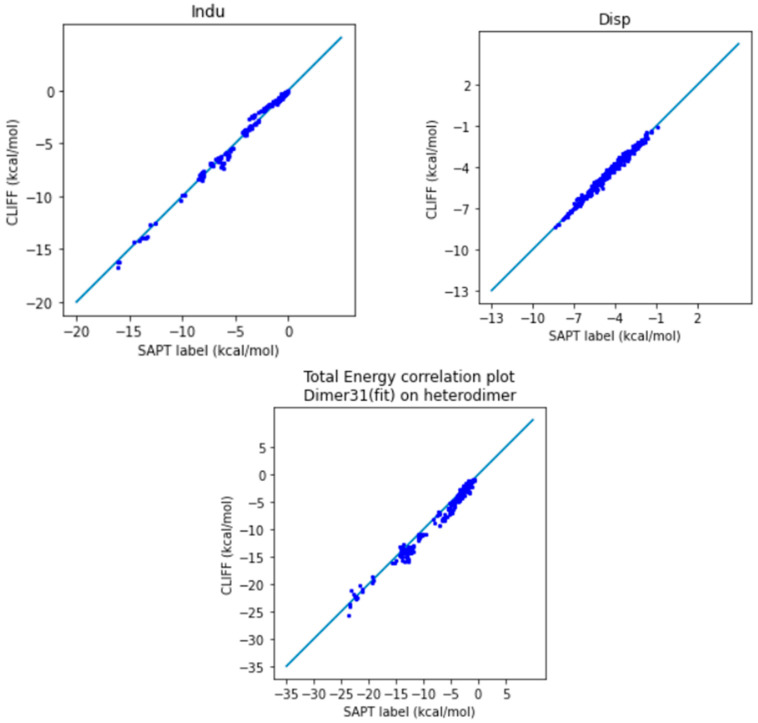
Correlation plots for calculating the energies of the SOFG-31-heterodimer using the SOFG-31 dataset as the training set. The blue line is the reference line for the correlation.

In Figure 2, we show the correlation plots for the predicted energies with the four SAPT energy components and the total energy calculated by the SAPT0 method (Figure 2). A closer alignment of the results to the diagonal reference line indicates a greater accuracy. We see that the overall distribution of the predicted component energy data are aligned along the reference data. In particular, the general accuracy for the van der Waals bounded portion of dimers is higher than the hydrogen bonded dimers. 

It is notable that the MAE for the total energy is 0.688 kcal/mol, which is well below the general standard of 1 kcal/mol. Moreover, from the correlation plots, the overall data distribution closely aligns with the reference line. This indicates that the parameters obtained from the SOFG-31 dataset perform well in predicting the energy data within the SOFG-31-heterodimer dataset. Despite the training dataset containing only homodimer data at equilibrium positions, it is worth exploring why it can accurately predict heterodimer interactions. This success can be attributed to the fact that in the model where interaction energies are summed over atomic pairs, the homodimers inherently provide the necessary information for calculating heterodimer interactions. Therefore, in this test case, we have demonstrated that the ML potential can do a good job interpolating the heterodimer interactions using homodimer energies as the training data.

Our results partially achieved our objectives. We selected homodimers with smaller carbon numbers from a set of fundamental functional groups as the fitting dataset. Following the fitting conditions, similar to those using the CLIFF0 dataset, the obtained parameters exhibit a strong predictive performance for the SOFG-31-heterodimer dataset. However, the currently re-fitted parameters encompass only a subset of atomic species. To accommodate a wider range of dimers, we will supplement the fitting dataset with homodimers from the Des370k set, thereby encompassing 17 different atomic species.

### 2.2. Employing the Dimer 31 + 47 as the Fitting Dataset

The Des370k dataset was constructed using a methodology similar to the SOFG-31 set, while it contains, as the name indicates, a large number of dimeric complexes. Compared with the SOFG-31 set, the Des370k includes twelve more organic functional group types: Esther, Ether, Nitrile, Sulfide, Disulfide, Amine, Cyclic Ether, Haloalkanes, Cyclic Sulfide, non-Aromatic Ring, Aromatic Ring, and Aromatic Secondary Amine; and the total dimer interaction energy data increase up to about 5000. It can be seen as an extension of the SOFG-31 dataset by extending the molecular types in a systematic way to more complex organic functional groups. Therefore, it is very useful to see the performance of the ML potential constructed based on these data. 

As can be seen in Table 2, the SOFG-31 dataset lacks some functional groups containing atoms such as nitrogen (N), sulfur (S), and halogens (F, Br, Cl), and from Table 1 we see we also need circular/ring/aromatic molecules. Therefore, we include these dimers from the Des370k set to compose the fitting set. These dimer data are also homodimers in the equilibrium position, similar to the SOFG-31 dataset. We select 47 homodimers at equilibrium positions from the Des370k dataset in order to supplement the SOFG-31 set in a new training set called the Dimer 31 + 47 set. These 47 dimers are from 2 Esters, 4 Ethers, 3 Nitriles, 5 Sulfides, 2 Disulfides, 5 Amines, 1 Cyclic Ether, 3 Haloalkanes, 1 Cyclic Sulfide, 9 Non-Aromatic Rings, 10 Aromatic Rings, and 2 Aromatic Secondary Amines. 

**Figure 3:**
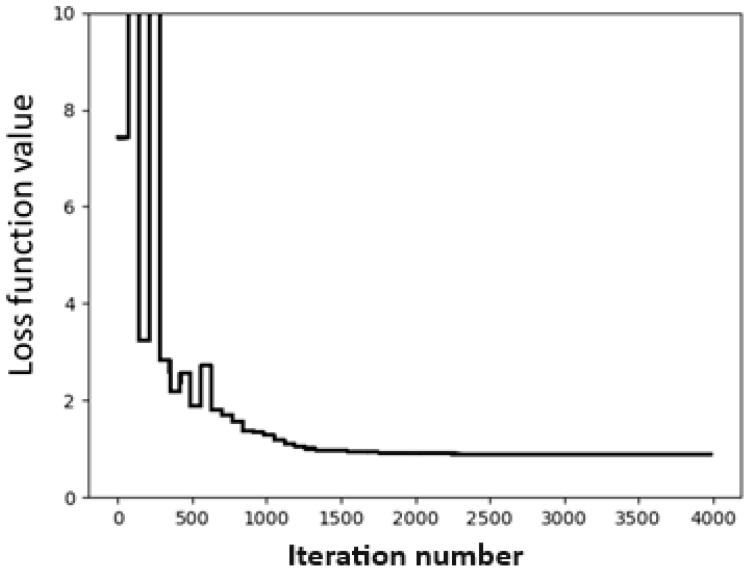
Convergence of loss function in the fitting process for the Dimer 31 + 47 dataset.

In this case, we use the Dimer 31 + 47 dataset as the training set and the SOFG-31-heterodimer dataset as the test set. For the convergence test, we use the same fitting conditions as before, namely the BFGS method and *γ* = 0.4. The convergence test is shown in Figure 3. It can be seen from Figure 3 that the fitting converged very quickly. Using the fitting, we obtain a set of global parameters, as shown in Table 4. It can be seen that the fitting set now contains all 17 atomic species.

#### 2.2.1. Predicted Results and Comparison to the SOFG-31-Heterodimer Dataset

As a test of the ML potential data, we employ the parameters derived from the Dimer 31 + 47 set, the training dataset, to predict the energy data within the SOFG-31- heterodimer dataset (Table 5). Here, we also compile the data using three measures: the mean absolute error (MAE), the root mean square error (RMSE), and the maximum deviation (MAX) for each SAPT energy component. As can be seen in Table 5, both the L1 and L2 error measures are within about 1 kcal/mol, which indicates the feasibility of the trained ML potential. The MAX values are well controlled, albeit a large value appears to be associated with the exchange repulsion energy, where the sampling points are rare. In Table 5, we see that the MAE of the total energy is 0.933 kcal/mol at this point. While it still is below the 1 kcal/mol threshold, there has been a slight increase compared to the previous MAE 0.688 kcal/mol. This increase can be attributed to the possibility of overfitting, due to the additional diversity of functional groups in the fitting dataset, which is lacking in the test set. As a result, there is a rise in the error for predicting this dataset containing only 31 molecules with 8 functional groups. This observation suggests that just blindly expanding the breadth or quantity of training data in the fitting does not necessarily lead to improved prediction results.

**Figure 4:**
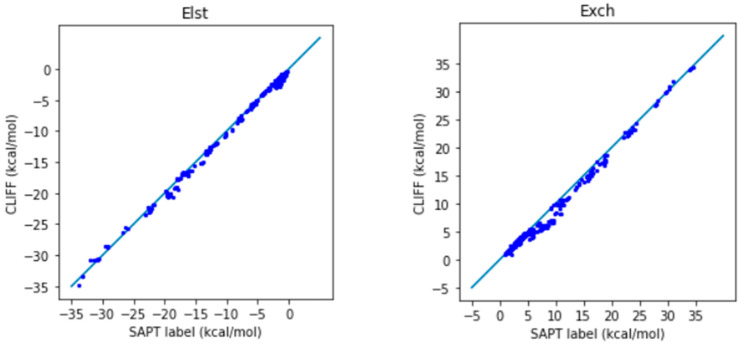

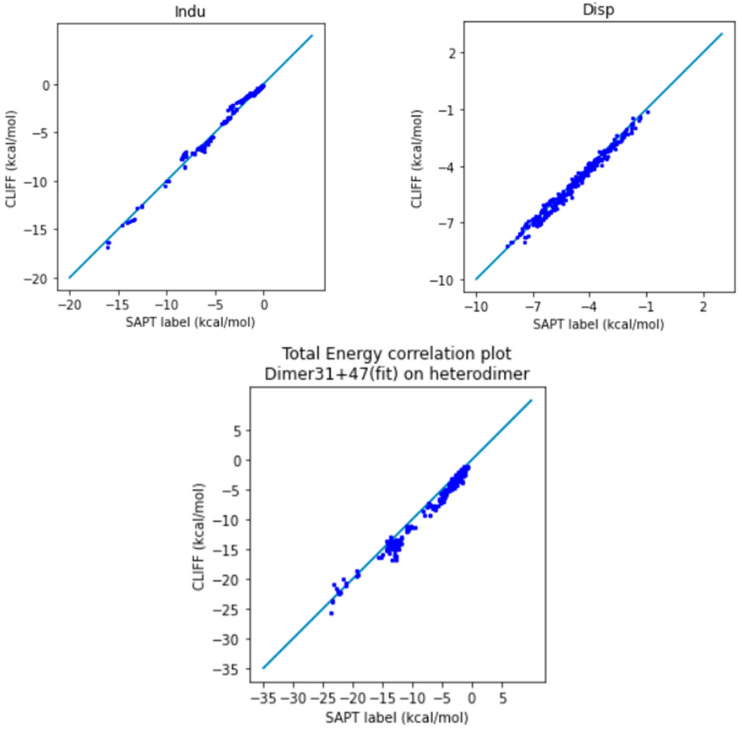
Correlation plots for calculating SOFG-31-heterodimer using Dimer 31 + 47 as the fitting dataset. The blue line is the reference line for the correlation.

In Figure 4, we show the correlation plots of the predicted energies with the four SAPT energy components and the total energy calculated using the SAPT0 method. A closer alignment of the results to the diagonal line indicates a greater accuracy. We see again that the overall distribution of the predicted data is aligned along the reference data. In particular, the general accuracy for the van der Waals bounded portion of dimers is higher than the hydrogen-bonded dimers. 

#### 2.2.2. Predicted Results and Comparison to the Des370k Dataset

**Figure 5:**
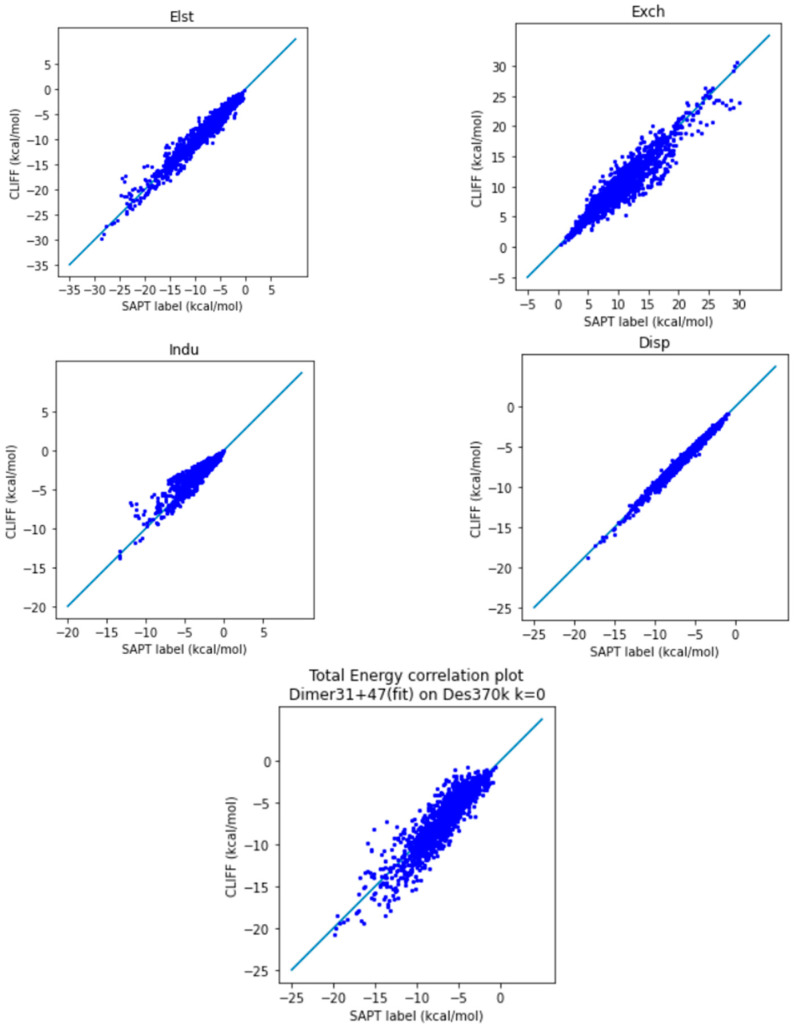
Correlation plot for calculating Des370k at k = 0 using Dimer 31 + 47 as the fitting dataset. The blue line is the reference line for the correlation.

**Figure 6:**
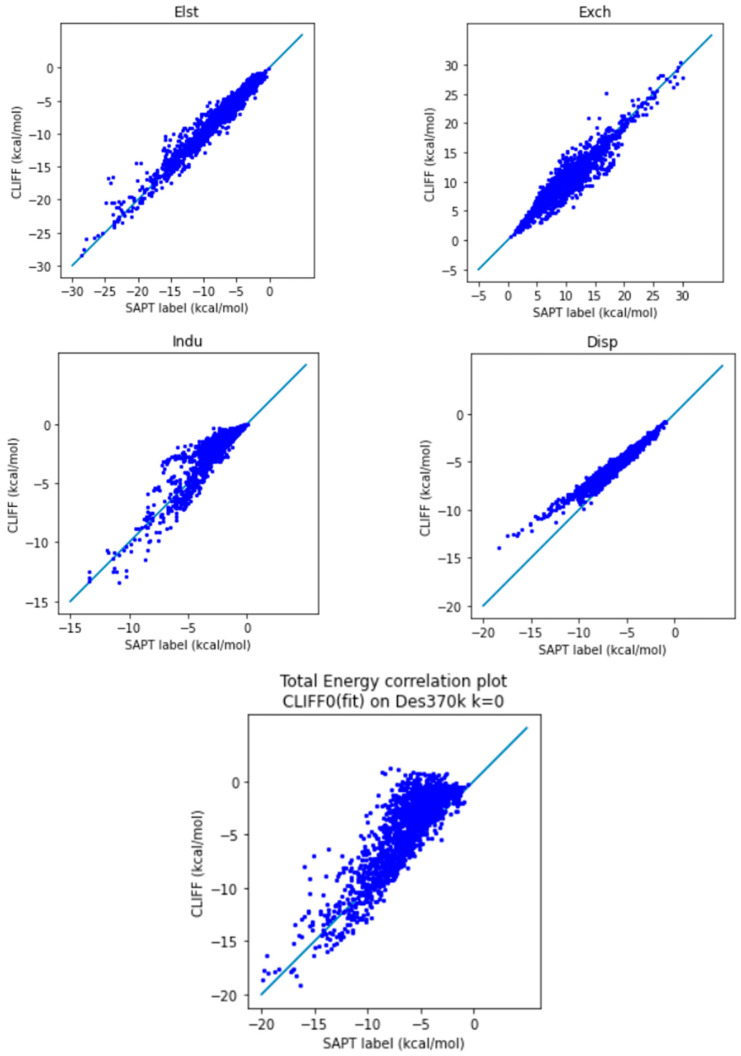
Correlation plot for calculating Des370k at k = 0 using CLIFF0 as the fitting dataset. The blue line is the reference line for the correlation.

In order to test the predictive ability of the constructed ML potential, we employ the parameters derived from the Dimer 31 + 47 set, the training dataset, to predict the energy data within the Des370k dataset. For comparison, we also perform a parallel series of modelling using the original CLIFF0 ML potential. Our results and the CLIFF0 results are shown in Table 6 and Table 7, and Figure 5 and Figure 6, respectively. As can be seen in Table 6, the L1 error measure is within about 1 kcal/mol, while the L2 measure is higher than 1 kcal/mol. This indicates that the predicted energy data are bias-distributed. Nonetheless, we see the MAX values are still well controlled, which indicates that there is error cancelling among the SAPT energy components. Indeed, as we can see from Figure 5, the exchange energy is normally underestimated, while the electrostatic and induction energies are overestimated. The sum of the all the energy components results in an MAE of the total energy being 0.979 kcal/mol at this point. 

On the other hand, the CLIFF0 results (Table 7 and Figure 6) exhibit worse results when compared to ours. Firstly, the MAE for the total energy is 2.108 kcal/mol; that is significantly larger than the chemical accuracy accepted. As can be seen from Table 7, the source of such a larger error comes from the induction and dispersion components. Both the L1 and L2 errors for these two components are significantly larger than ours. When examining the correlation plots for the individual energy components (Figure 6), our results in general outperform the CLIFF0 results in terms of induction energy (Indu) and dispersion energy (Disp), contributing to a smaller overall MAE. Indeed, as we can see from Figure 6, while the exchange energy is normally distributed, the induction and dispersion energies are significantly overestimated. In summary, we have demonstrated a superior error performance of the ML potential using our Dimer 31 + 47 set as the training set in testing the Des370k set, the dataset with the most diverse and numerous sets of molecules presently available. 

From these results, it is clear that utilizing the data structures and features of the training set helps in improving the predictive power of the trained ML potentials. Because both the SOFG-31 and Des370k datasets arrange the dimer data in a systematic and organized way, the trained ML parameters are more universally applied than by using a random set such as the CLIFF0 dataset. This specific point has not been systematically studied in constructing ML potentials and requires more exploration. Our results indicate that more organized data structures yield more reliable predictions.

## 3. Materials and Methods

The SOFG-31 dataset contains 8 common organic functional groups, namely alkanes, alkenes, alkynes, alcohols, aldehydes, ketones, amines, and acids, with a total of 31 homodimers at equilibrium. The basis set superposition error-corrected super-molecule approach using the second order Møller–Plesset perturbation theory (MP2) with Dunning’s aug-cc-pVXZ (X = D, T, Q) basis sets has been employed in the geometry optimization and energy calculations. The MP2 calculated interaction energies have been calibrated by using single-point calculations with the coupled cluster with single, double, and perturbative triple excitations method at the complete basis set limit [CCSD(T)/CBS] using well-tested extrapolation methods. We refer to alkanes, alkenes, and alkynes collectively as the AAA group, while alcohols, aldehydes, and ketones are designated as the AAK group, and carboxylic acids and amides are grouped as the CAA group. The SOFG-31-heterodimer dataset consists of dimeric interaction energy data derived from heterodimers formed by the monomers in the SOFG-31 dataset. The Des370k dataset was constructed using a methodology similar to the SOFG-31 set, while it includes twelve more organic functional group types: Esther, Ether, Nitrile, Sulfide, Disulfide, Amine, Cyclic Ether, Haloalkanes, Cyclic Sulfide, non-Aromatic Ring, Aromatic Ring, and Aromatic Secondary Amine. Both homodimers and heterodimers are included in the Des370k dataset. The CLIFF0 dataset, however, contains dimers selected from public databases, and the data have not been classified according to specific molecular types.

The SAPT energy is divided into four components: electrostatic (Elst), exchange–repulsion (Exch), dispersion (Disp), and induction (Indu) energies. The CLIFF scheme models these four components using equations that rely on electronic density overlaps. In order to formulate these equations in a pairwise-atomic manner, it is necessary to partition the electron densities of the monomers from their constituent atoms. This partitioning is accomplished through the utilization of the atoms-in-molecules (AIM) method. The AIM densities obtained in this process represent the atom-centered electronic distributions that inherently take into account their local chemical surroundings. This approach provides a viable means to develop accurate and transferable models. The CLIFF scheme makes use of atomic multipoles, atomic widths, and the Hirshfeld ratios as atomic properties, all of which can be computed from the AIM densities. 

For the calculation of atomic multipoles, atomic widths, and Hirshfeld ratios, the CLIFF employs distinct machine learning models for each chemical element, including C, H, N, O, S, F, Cl, and Br, resulting in a total of 24 models. In all cases, these models rely on a kernel-ridge regression (KRR) approach. The database used exclusively comprises structures sourced from the ChEMBL database. To create this database, the CLIFF applied a filtering process to select a subset of approximately 872,000 drug-like molecules. Given that the atomic properties computed are inherently local in nature, the CLIFF opts to fragment this dataset into molecules containing 5 to 12 heavy atoms. Subsequently, by curating unique fragments from this fragmentation, it results in a collection of 8,138 chemically diverse molecules. These molecules possess structural moieties representative of drug-like compounds and protein targets.

To obtain the reference densities, the CLIFF utilized the PBE0/aug-cc-pV(D+d)Z method using Psi4 software (version 1.3). Reference atomic properties were determined using the MBIS and Hirshfeld routines, which are implemented in Horton software (version 2.1.1). Here we summarize the mathematical equations for the four energy components; details can be found in the CLIFF paper [45].

### 3.1. Electrostatics

The electrostatic energy model employed is the Damped Multipole Electrostatic model. This model takes into account the forces between atomic nuclei within atomic pairs, the forces between atomic nuclei and multipole moments, and the interactions among different multipole moments.

(2) $$E_{elst}=\sum_{i\in A}^{}\sum_{j\in B}^{}\frac{Z_{i}Z_{j}}{r_{ij}}+Z_{i}T_{ij}{}^{f_{1}}M_{j}+M_{i}{}^{T}T_{ij}{}^{f_{1}}Z_{j}+M_{i}{}^{T}T_{ij}{}^{f_{2}}M_{j}$$

These *T* matrices, respectively, represent the damping interaction tensors between atomic nuclei and multipole moments, as well as between different multipole moments. The damping functions are defined as

(3) $$f_{1}(r_{ij})=1-e^{-K_{i}{}^{elst}r_{ij}}$$

(4) $$f_{2}(r_{ij})=1-\frac{{\left(K_{i}{}^{elst}\right)}^{2}}{{\left(K_{i}{}^{elst}\right)}^{2}-{\left(K_{j}{}^{elst}\right)}^{2}}e^{-K_{i}{}^{elst}r_{ij}}-\frac{{\left(K_{j}{}^{elst}\right)}^{2}}{{\left(K_{j}{}^{elst}\right)}^{2}-{\left(K_{i}{}^{elst}\right)}^{2}}e^{-K_{j}{}^{elst}r_{ij}}$$

where the fitting parameters $K_{i}{}^{elst}$ are required to be determined using the ML scheme. The values may differ based on the specific types of atoms they correspond to. The fitting process is conducted using the electrostatic energies obtained from the SAPT calculations.

### 3.2. Exchange–Repulsion

In the model, exchange energy is described as the repulsive force arising from the overlap of electron densities between pairs of atoms.

(5) $$E_{exch}=\sum_{i\in A,j\in B}^{}K_{ij}{}^{exch}S_{ij}$$

Here, $K_{i}{}^{exch}$ correspond to different atomic species, and the fitting will be carried out using the exchange energies computed from the SAPT calculations. The *S* matrices are calculated as follows:

(6) $$B_{ij}=\frac{1}{\sigma_{i}\sigma_{j}}$$

(7) $$S_{ij}=[\frac{1}{3}{\left(B_{ij}r_{ij}\right)}^{2}+B_{ij}r_{ij}+1]e^{-B_{ij}r_{ij}}$$

### 3.3. Dispersion

Dispersion energy is based on attractive forces generated by atomic polarization and interactions with electrons. Here, the Tang–Toennies damped dispersion model is employed. First, coefficients for atom pairs are calculated:

(8) $$C_{6,ij}=-\frac{2C_{6,i}C_{6,j}}{\frac{\alpha_{i}}{\alpha_{j}}C_{6,i}+\frac{\alpha_{j}}{\alpha_{i}}C_{6,j}}$$

(9) $$C_{6,i}=C_{6,i}^{free}h_{i}{}^{2},\alpha_{i}=\alpha_{i}^{free}h_{i}$$

where h represents the Hirshfeld ratios calculated from the machine learning model, $C_{6,i}$ stands for the monomer coefficients, and $\alpha_{i}$ represents the atomic polarizability. The value $\alpha_{{}_{i}}^{free}$ is determined using calculations based on the free atomic density. To calculate $C_{8,ij}^{}$, we use

(10) $$C_{8,ij}=3C_{6,ij}\sqrt{Q_{i}Q_{j}},Q_{i}=\sqrt{Z_{i}}\frac{\langle r_{i}^{4}\rangle }{\langle r_{i}^{2}\rangle }$$

where $\langle r_{i}^{n}\rangle$ is the multipole expectation value that can also be calculated from the atomic density. To calculate $C_{10,ij}$, we use

(11) $$C_{10,ij}=\frac{49}{40}\frac{C_{8,ij}^{2}}{C_{6,ij}}$$

The Tang–Toennies damping function is

(12) $$f_{n}=1-(\sum_{k=0}^{n}\frac{x_{ij}^{k}}{k!})e^{-x_{ij}},x_{ij}=B_{ij}r_{ij}+\frac{2B_{ij}^{2}+3B_{ij}}{{\left(B_{ij}r_{ij}\right)}^{2}+3B_{ij}r_{ij}+3}r_{ij}$$

Finally, we obtain the dispersion energy

(13) $$E_{disp}=\sum_{i\in A}^{}\sum_{j\in B}^{}\left(\frac{C_{6,ij}}{r^{6}}f_{6}(r_{ij})+K_{ij}^{disp}\sum_{n=8,10}^{}\frac{C_{n,ij}}{r^{n}}f_{n}(r_{ij})\right)$$

### 3.4. Induction

Induction energy refers to the interaction energy generated by the polarization of atoms due to the electric field of another atom. Here, the Thole method is employed.

(14) $$E_{ind}=\sum_{i\in A}^{}\sum_{j\in B}^{}\mu_{i}^{\prime }T_{ij}M_{j}+K_{ij}^{indu}S_{ij}$$

where $\mu_{i}^{\prime }$ is the induced atomic dipole, derived through the iteration,

(15) $$\mu_{i}^{\prime }(n+1)=(1-\omega )\mu_{i}^{\prime }(n)+\omega [\mu_{i}^{\prime }(0)+\alpha_{i}\sum_{\begin{array}{l} k\in A\cup B\\ k\neq i \end{array}}^{}T_{ik}M_{k}]\;\mu_{i}^{\prime }(0)=\alpha_{i}\sum_{j\in B}^{}T_{ij}M_{j}$$

where *k* ranges over all atoms except *i* in the dimer, with ω = 0.7. The interaction tensor, $T_{ij}$, uses the Thole damping to smear atomic charge distributions.

(16) $$f_{Thole}=\frac{3a}{4\pi }e^{-au^{3}},u=r_{ij}/{\left(\alpha_{i}\alpha_{j}\right)}^{\frac{1}{6}}$$

where a is the smearing coefficient conventionally defined as 0.39.

## 4. Conclusions

We performed a machine learning test on the recently proposed CLIFF kernel type modeling of intermolecular non-bonded interactions. The training data were from the SOFG-31 dimer dataset and the Des370K dataset. We deliberately built our ML potentials by using the designated features of these datasets, namely, the well-arranged organic functional groups and a systematic inclusion of analogous dimers in the training sets. Three tests were performed: (1) Training the SOFG-31 homodimer to test the SOFG-31+269 heterodimer sets, with an overall MAE of 0.688 kcal/mol. (2) Training the Dimer 31 + 47 to test the SOFG-31+269 heterodimer sets, with an overall MAE of 0.933 kcal/mol. (3) Training the Dimer 31 + 47 to test the Des370k sets, with an overall MAE of 0.979 kcal/mol. Our results clearly show that by using a systematic construction of training datasets one can predict a wide range of interaction patterns and energies. It is thus very promising that machine learning techniques are useful and feasible in force field modelling.

## Figures and Tables

**Table 1:** The SOFG-31 dataset.

Alkane	Alkene	Alkyne	Alcohol	Aldehyde	Ketone	Acid	Amide
Methane	Ethylene	Ethyne	Methanol	Formaldehyde	Acetone	Formic acid	Formamide
Ethane	Propylene	Propyne	Ethanol	Acetaldehyde	Butanone	Acetic acid	Acetamide
Propane	Butylene	Butyne	Propanol	Propanaldehyde	Pentanone	Propanoic acid	Propanamide
Butane	Pentylene	Pentyne	Butanol	Butanal			
Pentane							
Hexane							

**Table 2:** The CLIFF Parameters of the fitting set SOFG-31.

	$K^{elst}$	$K^{exch}$	$K^{indu}$	$K^{disp}$
C4	3.039	2.313	0.580	0.194
C3	2.996	2.594	1.040	0.373
C2	3.030	2.724	1.266	0.730
N3	3.088	4.911	1.596	0.245
N2	-	-	-	-
N1	-	-	-	-
O2	5.309	4.508	1.547	0.098
O1	3.829	4.022	2.487	0.702
S2	-	-	-	-
S1	-	-	-	-
HC	4.755	0.881	0.180	0.003
HN	3.095	0.925	0.320	0.001
HO	2.619	0.769	0.351	0.003
HS	-	-	-	-
F	-	-	-	-
Cl	-	-	-	-
Br	-	-	-	-

**Table 3:** Using the SOFG-31 set to predict the SOFG-31-heterodimer results.

SOFG-31 (Fit)	MAE	RMSE	MAX
Elst	0.403	0.517	2.081
Exch	0.626	0.912	2.777
Indu	0.204	0.314	1.180
Disp	0.150	0.196	0.555
Total	0.688	0.971	3.177

Energy in kcal/mol.

**Table 4:** Parameters obtained using Dimer 31 + 47 as the fitting dataset.

	$K^{elst}$	$K^{exch}$	$K^{indu}$	$K^{disp}$
C4	3.306	1.521	0.326	0.118
C3	3.185	2.517	0.825	0.558
C2	3.136	2.859	0.918	0.547
N3	3.764	3.228	0.898	0.557
N2	3.372	3.443	1.637	0.331
N1	3.308	2.996	1.215	0.515
O2	4.402	4.704	1.019	0.396
O1	3.616	4.073	2.180	0.219
S2	3.198	2.764	1.125	0.648
S1	3.075	3.061	0.979	0.424
HC	3.443	1.186	0.429	0.216
HN	2.855	1.047	0.454	0.011
HO	2.784	0.754	0.434	0.0003
HS	3.218	1.334	0.78	0.042
F	3.935	6.683	1.217	0.001
Cl	3.367	3.444	0.761	0.472
Br	3.734	3.734	0.385	3.734

**Table 5:** Results of calculating SOFG-31- heterodimer using Dimer 31 + 47 as the fitting dataset.

Dimer 31 + 47	MAE	RMSE	MAX
Elst	0.383	0.506	2.058
Exch	0.688	0.990	2.913
Indu	0.213	0.340	1.169
Disp	0.186	0.232	0.714
Total	0.933	1.169	3.916

Energy in kcal/mol.

**Table 6:** Results of calculating Des370k at equilibrium point using Dimer 31 + 47 as the dataset.

Dimer 31 + 47	MAE	RMSE	MAX
Elst	0.723	1.002	7.001
Exch	1.076	1.542	6.431
Indu	0.447	0.724	5.287
Disp	0.236	0.296	1.310
Total	0.979	1.342	6.977

Energy in kcal/mol.

**Table 7:** Results of calculating Des370k at equilibrium using CLIFF0 as the fitting dataset.

CLIFF0	MAE	RMSE	MAX
Elst	0.762	1.058	7.863
Exch	1.115	1.519	8.261
Indu	0.697	0.927	3.957
Disp	0.904	1.124	4.802
Total	2.108	2.605	9.516

Energy in kcal/mol.

## Data Availability

The data that supports the findings of this study are available within the article and can be obtained from the authors.
